# Supplementary material for: Acceleration of FM-index Queries Through Prefix-free Parsing
Source: Algorithms Bioinform. Author manuscript; Available in PMC 2025 Nov 1. (PMC12576618; doi:10.4230/LIPIcs.WABI.2023.13)
Supplement: Supplement, including Figs 6--8 [file NIHMS2112388-supplement-Supplement__including_Figs_6--8.pdf]

A Appendix

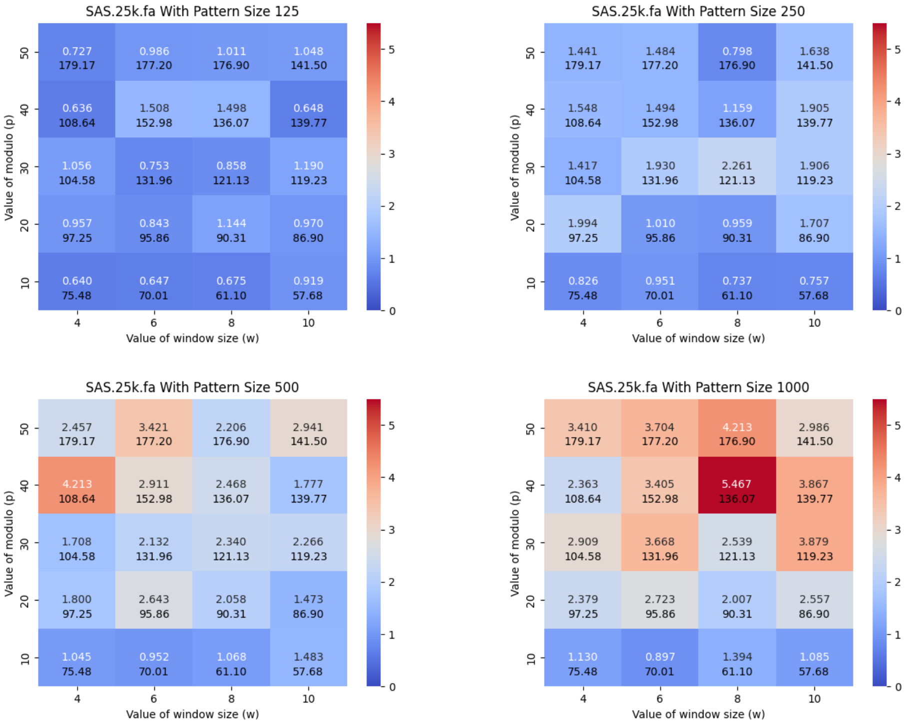

Figure 6 Illustration of the impact of w, p and the length of the query pattern on the acceleration of the FM-index. Here, we used `SARS-25K` dataset and varied the length of the query pattern to be equal to 125, 250, 500, and 1000. The y-axis corresponds to p and the x-axis corresponds to w. The heatmap illustrates the number of queries that can be performed in a CPU second with the acceleration verses the standard FM-index from `sdsl`, i.e., `PFP-FM` / `sdsl`.

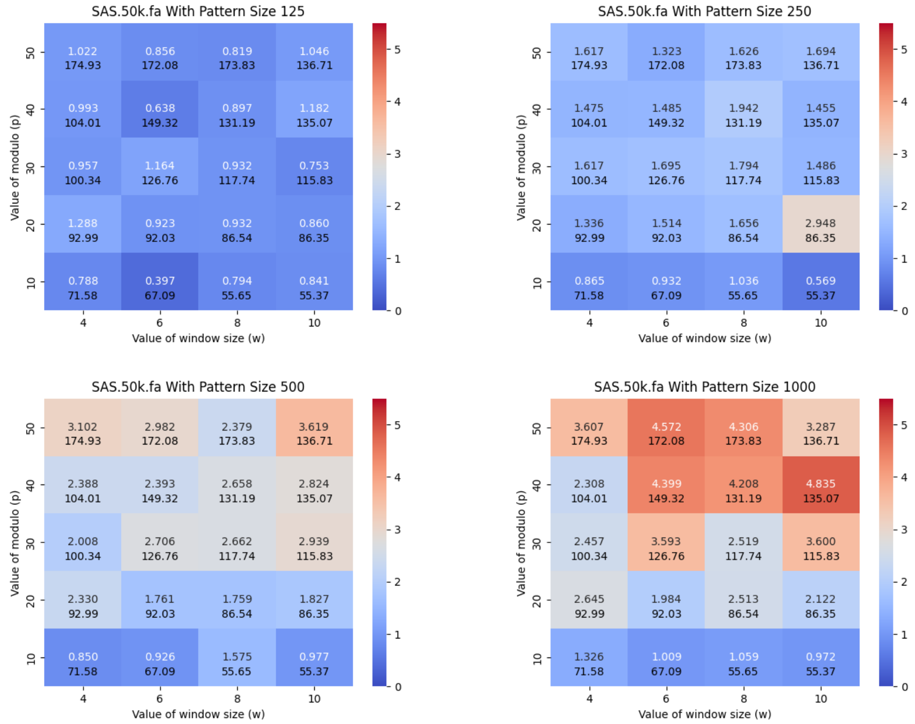

Figure 7 Illustration of the impact of w, p and the length of the query pattern on the acceleration of the FM-index. Here, we used `SARS-50K` dataset and varied the length of the query pattern to be equal to 125, 250, 500, and 1000. The y-axis corresponds to p and the x-axis corresponds to w. The heatmap illustrates the number of queries that can be performed in a CPU second with the acceleration verses the standard FM-index from `sdsl`, i.e., `PFP-FM` / `sdsl`.

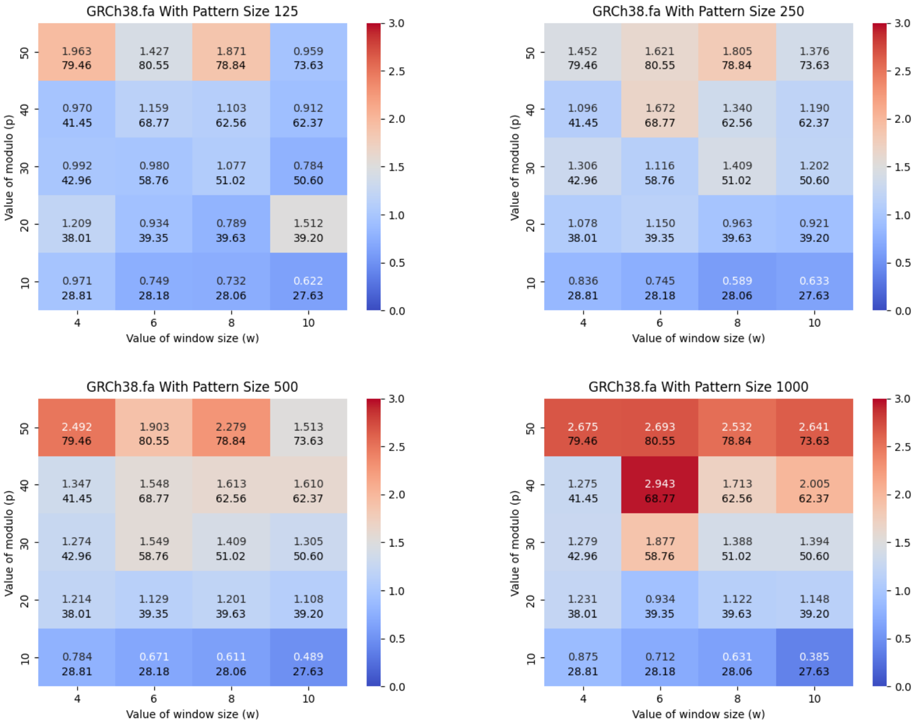

Figure 8 Illustration of the impact of w, p and the length of the query pattern on the acceleration of the FM-index. Here, we used GRCh38 dataset and varied the length of the query pattern to be equal to 125, 250, 500, and 1000. The y-axis corresponds to p and the x-axis corresponds to w. The heatmap illustrates the number of queries that can be performed in a CPU second with the acceleration verses the standard FM-index from sdsl, i.e., PFP-FM / sdsl.
