# Supplementary figures and images for: Acceleration of FM-index Queries Through Prefix-free Parsing
Source: Algorithms Bioinform. Author manuscript; Available in PMC 2025 Nov 1. (PMC12576618; doi:10.4230/LIPIcs.WABI.2023.13)

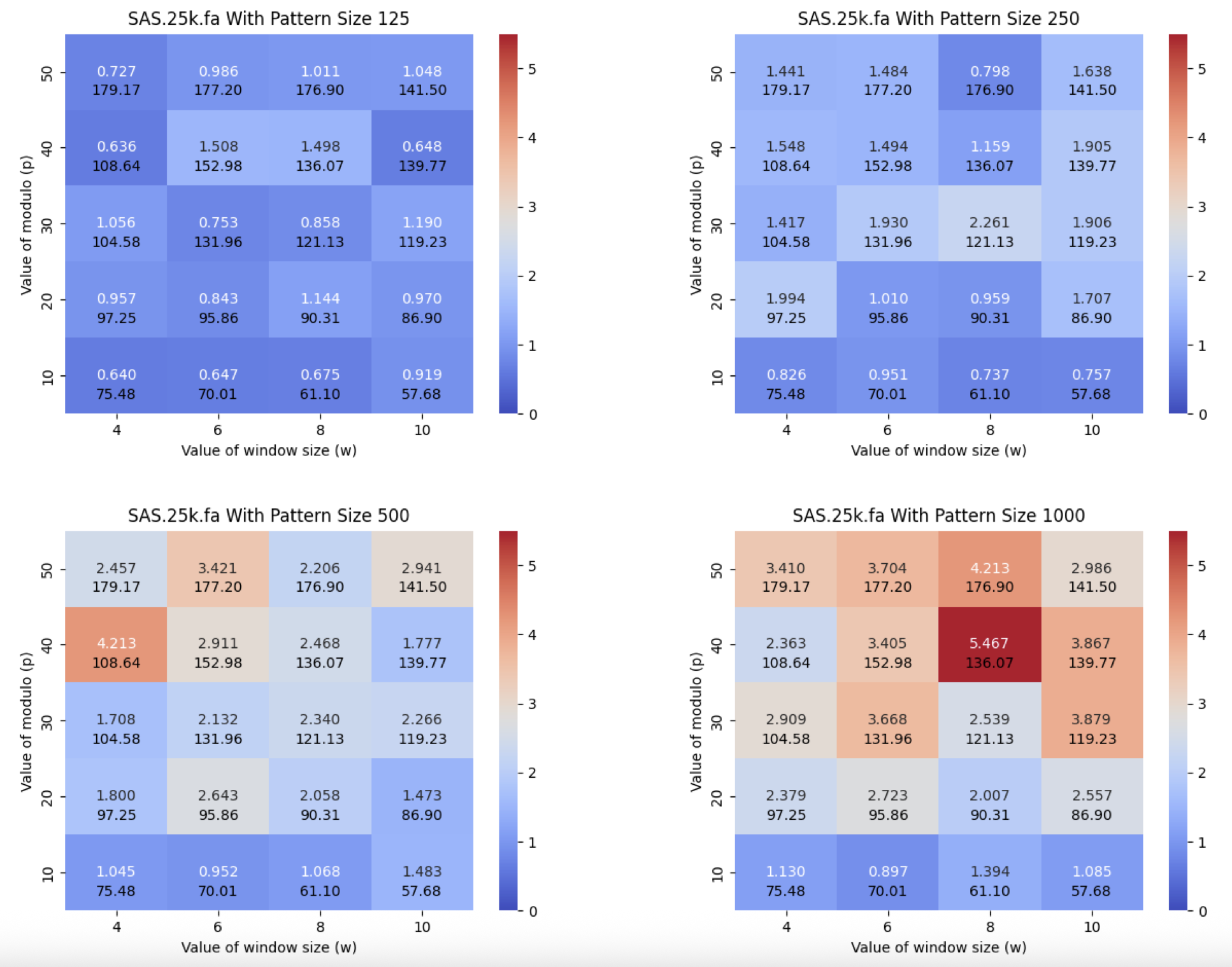

Supplement: 1 [file NIHMS2112388-supplement-1.png]

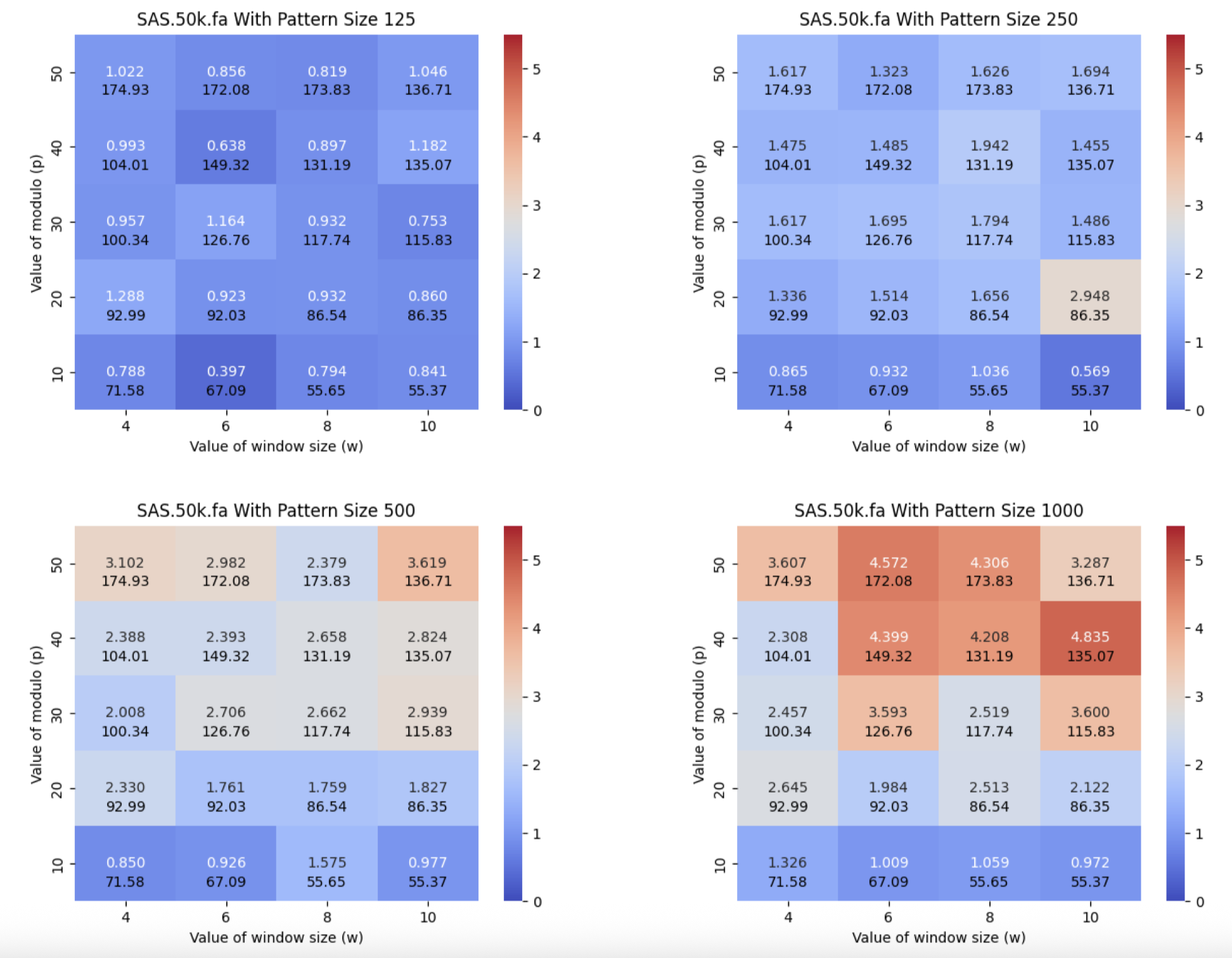

Supplement: 2 [file NIHMS2112388-supplement-2.png]

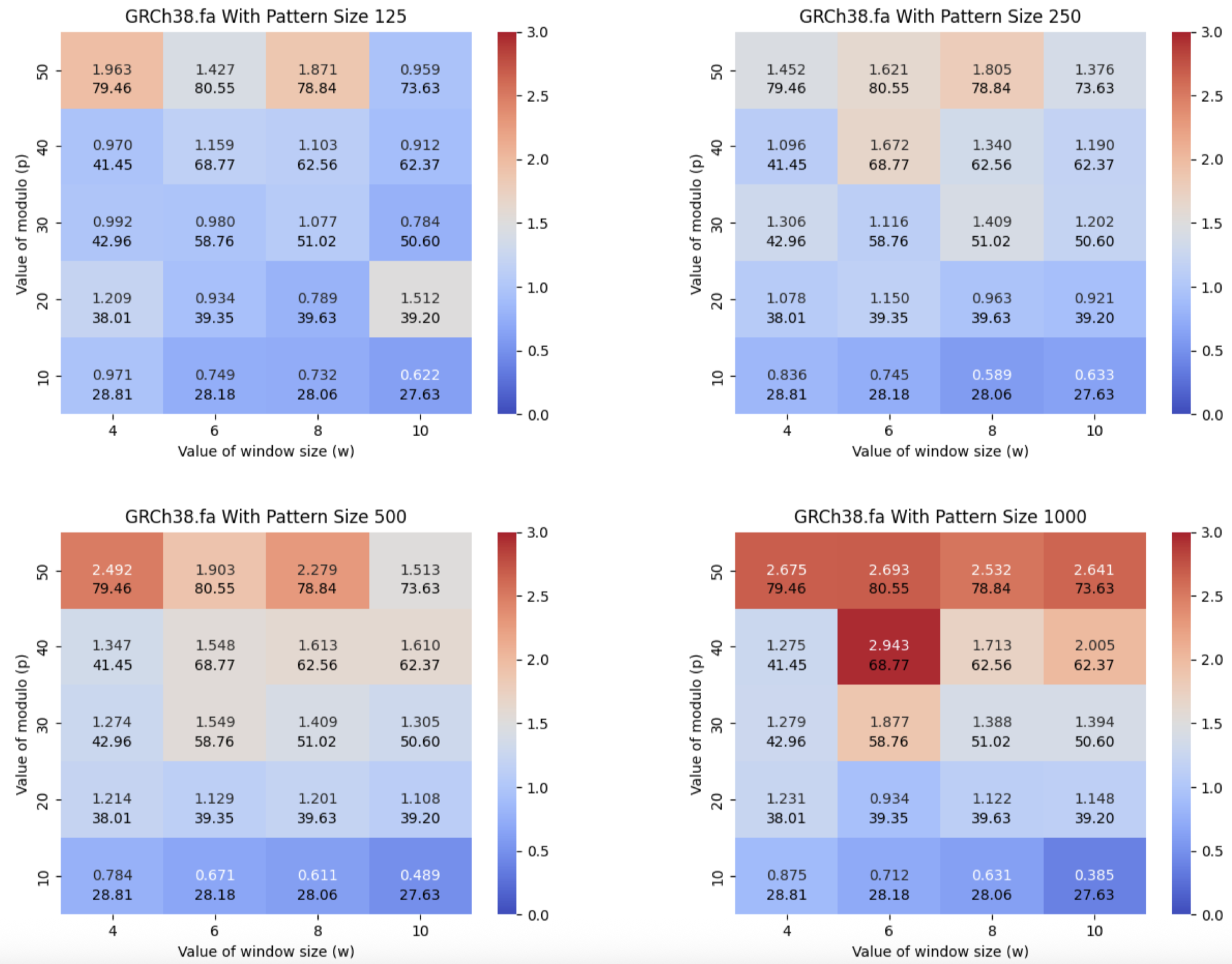

Supplement: 3 [file NIHMS2112388-supplement-3.png]
